# Online student tutorials for effective peer teaching in digital times: a longitudinal quantitative study

**DOI:** 10.1186/s12909-022-03741-9

**Published:** 2022-09-16

**Authors:** Teresa Festl-Wietek, Nils Kern, Rebecca Erschens, Jan Griewatz, Stephan Zipfel, Anne Herrmann-Werner

**Affiliations:** 1 TIME - Tübingen Institute for Medical Education, University of Tuebingen, Tuebingen, Germany; 2 Deanery of Students’ Affairs, University of Tuebingen, Tuebingen, Germany; 3 Department of Psychosomatic Medicine and Psychotherapy, University Hospital Tuebingen, Tuebingen, Germany

**Keywords:** Online student tutorials, Peer-assisted learning, Nonverbal communication, Interaction, Doctor-patient communication

## Abstract

**Background:**

Peer-assisted learning represents a favoured method of teaching in universities. The COVID-19 pandemic has necessitated transferring medical education to digital formats, and subsequently, the question has arisen of whether online tutorials might be effective. This study, thus, investigated the efficacy of online tutorials in a communication course by assessing the interaction, verbal communication, and nonverbal communication of tutors and students.

**Methods:**

Second-year medical students were invited to participate in this longitudinal quantitative study. Validated and self-developed questionnaires (e.g., Jefferson Empathy Scale) including 39 questions (rated on a 7- or 5-point Likert scale) were used to assess the different variables including interaction, verbal and nonverbal communication and students’ learning success.

**Results:**

Out of 165 medical students, 128 took part in the study. The students as well as tutors reported that they found each other likeable (M_students_ = 4.60±0.71; M_tutors_ = 4.38±0.53; *p* > .05). Learning success increased throughout the communication course (Cohen’s *d* = 0.36–0.74). The nonverbal and verbal communication in the simulated patient (SP) encounter was also rated as high by all three groups (M_nonverbal_ = 3.90±0.83; M_verbal_ = 4.88±0.35).

**Conclusions:**

Interaction as well as nonverbal and verbal communication occurred in the online format, indicating that online tutorials can be effective. The implementation of SPs increases the efficiency of synchronous online learning as it enhances the simulation of a real patient–physician encounter. Thus, online tutorials are a valuable amendment to medical education.

## Introduction

Peer-assisted learning (PAL) represents a favoured method of teaching in universities worldwide, with medical students typically being taught by their peers [1]. As a traditional teaching concept, PAL is considered mutually beneficial as students learn from each other [2, 3]. Topping and Ehly (1998) strengthened the reduced distance learning in PAL so that students are taught by student tutors who previously took the course [4, 5]. In a traditional teacher–student relationship, the teachers and students are on different levels [4, 5]. In contrast, peers or student tutors are closer to the students, better understand their difficulties, and can explain the subject matter at an appropriate level [6–8]. Therefore, students and student tutors often have a trust-based relationship, which could be built through in-person interactions in pre-COVID times [9, 10].

### Online teaching

However, due to the COVID-19 pandemic, lessons in medical education could no longer be performed in the traditional face-to-face manner and needed to be transferred to digital formats; this is believed to impact the level of trust between the students and tutors. The reported challenges in online teaching settings included issues relating to communication, assessment, and lack of expertise with digital tools [11, 12]. Tutors argued that the private atmosphere of trust that is characteristically established by tutors would be negatively affected by an online format where interaction and communication are restricted, due to which the students might not feel free to ask questions without fear of judgement [13].

Medical students, however, could also see benefits and new opportunities in online education, including the integration of virtual reality formats [11, 12]. In a cross-sectional study in the UK, Dost et al. [14] suggested that the efficacy of medical education might be increased when traditional face-to-face teaching is combined with online teaching in team-based or problem-based learning. Subsequently, tutorials have also been transferred into digital synchronous teaching modules. In a study by Co et al. [15], medical students’ clinical competency in basic surgical skills was compared following different teaching methods, including in-person and online teaching. The results showed no significant difference in the clinical competency of students taught using the various methods.

Furthermore, Hodgson and Hagan (2020) realigned the tutorials into virtual settings to support social contact during the pandemic. They found that video calls were superior to audio calls in terms of the quality of the experience and the flow of conversation [16]. In addition, the scheduled video calls provided social support, which was otherwise highly reduced during the pandemic; therefore, they enhanced the students’ well-being. Thus, while tutorials might be effective in a digital format, the question arises as to which contributing factors (e.g., communication between students and tutors) might be preserved and which might be lost or diminished (e.g., empathy, trustful relationship).

### Aim

This study aimed to investigate the efficacy of online tutorials in a communication course by assessing the interactions and verbal and nonverbal communication between tutors and students. In addition, students’ learning success was assessed over the duration of the course. The study also investigated the students’ empathy in general as well as their empathic behaviour in a simulated patient–physician encounter online.

## Methods

### Study design

This study followed a longitudinal quantitative design in order to investigate the efficacy of an online student tutorial in a preclinical communication course. Second-year medical students were invited to take part. The study took place at the Medical Faculty of the Eberhard-Karls University of Tuebingen during the summer term (from April to July 2021).

### Ethics

The study received ethics approval from the Ethics Committee of Tuebingen Medical Faculty (no. 162/2021BO2). Participation was voluntary, but participation in the course was mandatory. Students did not receive any reimbursement for participating. All participants provided their written informed consent and their responses and data were kept anonymous.

### Measurements

Reliable and validated questionnaires were used to assess the relevant variables. The degree of empathy in patient-physician communication was measured using the Jefferson Scale of Physician Empathy, Student Version (JSPE-S) [17]. The JSPE-S consists of 20 items on a seven-point Likert scale ranging from 1 (strongly disagree) to 7 (strongly agree). The simulated patients (SP) completed the Jefferson Scale of Patient Perception of Physician Empathy (JSPPE) with five items ranging on the same scale.

Learning success was assessed based on the students’ knowledge of psychosocial medical history [18]. They were asked to complete a checklist in which they named those elements of patients’ psychosocial medical history that were relevant to their medical history. The reference points for the measurements were: the start of the communication course (T0), during the practice unit after the patient–physician encounter (T1), and the end of the communication course (T2). The interval considered was one week between T0 and T1 and 2–3 weeks between T1 and T2. This assessment took place online.

Furthermore, the level of interaction in the online tutorial was measured using items developed by the researchers. These items were rated on a five-point Likert scale ranging from 1 (strongly disagree) to 5 (strongly agree). There were three different versions, depending on the corresponding perspective (student, tutor, SP). An example of the student’s version was “the tutor used his knowledge to help us”. An example of the tutor’s version was “I used my knowledge to help the students”. For verbal and nonverbal communication, 10 items were developed by the researchers that were rated on the same scale as the interactions. One example was “the student was able to react adequately to patient’s verbal and nonverbal hints”. Students as well as student tutors filled in these items. The SP also completed 10 items developed by the researchers on the same scale but with different descriptions related to their encounter with the physician. One example was “the student told me relevant information in the right volume and in an understandable way”.

The participants were also asked about any technical difficulties that they faced (e.g., internet connection or video transmission issues) and to what degree they affected the SP–physician encounter.

### Procedure

This study was implemented in an existing basic communication course on taking medical histories with an increasing degree of fidelity (using role play and simulated and real patients). Due to the pandemic, the entire course took place online using the Zoom platform. The study took place in the second practice unit, which was taught by a student tutor. Here, the students had to record the psychosocial medical history of an SP suffering from poorly controlled diabetes and wearing an obesity simulation suit [19]. The students were expected to conduct the conversation in an empathic, easily understandable way and detect the psychosocial factors that explained why the patient was unable to handle her diabetes. At the end of the practice unit, all participants filled in the questionnaires.

For assessing learning success, the students were asked to name all the relevant elements of the psychosocial medical history at the three different time points discussed previously: T0, T1, and T2.

### Data analysis

The normal distribution of the data was verified using the Kolmogorov–Smirnov test. The descriptive statistics were calculated, including mean values (*M*), standard deviations (*SD*), sum scores, frequencies, and percentages of the relevant factors including nonverbal, verbal communication, interaction and technical difficulties. Any missing value was replaced with the mean value. T-Tests were conducted for the items associated to interaction, nonverbal and verbal communication. As the data was normally distributed we decided to use t-tests as parametric method. However, we doublechecked the results by using Mann-Whitney U test. Comparisons among all three groups, including students, tutors, and SPs, were performed using ANOVAs and the Scheffé post-hoc test. Learning success was compared by using *t*-tests for dependent samples over the different time points. Furthermore, correlations were determined by calculating Pearson and Spearman correlation coefficients. The Statistical Package for the Social Sciences version 26.0 (IBM, Armonk, NY, USA) was used for data analysis. The level of significance was set to *p <* .05.

## Results

### Sample

In all, 128 out of 165 students (RR = 77.6%) participated in the study. The students’ average age was 22.64±2.44 years. Of them, 67.7% were female. Five tutors participated in the study with an average age of 25.60±3.26 years. A total of 80.0% of the tutors were female. Eight SPs participated.

### Interaction

The students and tutors agreed that the degree of interaction was high in the online tutorial. They agreed that the tutors used their knowledge to help the students (M_students_ = 4.60±0.71; M_tutors_ = 4.38±0.53; *p* > .05). In addition, they found each other likeable and felt that the other group behaved empathically, with the students agreeing significantly more to this statement (M_students_ = 4.65±0.63; M_tutors_ = 4.33±0.86; *p* < .01). The students also reported that they could express their difficulties to the tutor (M = 4.03±0.87). This statement was confirmed by the tutors (M = 3.86±1.11; *p* > .5). Furthermore, the medical students agreed that they learned a great deal during the online tutorial (M_students_ = 3.76±1.02; M_tutors_ = 3.86±0.96; *p* > .05).

### General evaluation of the online format

The students tended to agree that the online format affected their attention level (M = 2.77±1.13). However, the tutors disagreed with the notion of the format affecting the students’ attention (M = 3.43±0.87; *p* < .01). Both groups disagreed that they preferred the online format in comparison to the face-to-face version (M_students_ = 2.05±1.17; M_tutors_ = 1.95±1.02; *p* > .05). However, they agreed that the online tutorial was very helpful (M_students_ = 3.82±0.97; M_tutors_ = 3.90±0.83; *p* > .05) and liked its presentation (M_students_ = 3.97±0.83; M_tutors_ = 3.71±1.10; *p* > .05). Further, the students indicated that they would recommend the online tutorial to their fellow students (M = 3.98±0.99).

### Nonverbal and verbal communication

Table 1 presents the items related to nonverbal and verbal communication delineated for the three groups: medical students, tutors, and SPs. Several significant differences can be observed among the three groups. Please see Table 1 for more details.

**Table 1:** Items related to nonverbal and verbal communication delineated for the three groups and investigation of possible significant group differences

Items	Medical student (groups 1)		Tutor (group 2)		Simulated patient (group 3)		Group difference	
The student …	M	SD	M	SD	M	SD	p	Scheffé
had eye contact	4.54	0.90	4.10	0.70	4.88	0.35	> .05	-
had a straight posture to signal that the issue is serious	4.38	0.94	4.05	0.81	4.75	0.71	> .05	-
used non-verbal cues, and used them deliberately	4.41	0.77	3.90	0.83	4.88	0.35	= .029 = .010	1-2 2-3
delivered relevant information in an understandable way	4.71	0.69	4.14	0.85	4.75	0.46	= .005	1-2
recognised the patient’s feelings based on nonverbal cues	4.20	0.86	3.76	1.09	4.63	0.52	> .05	
responded adequately to patient’s nonverbal and verbal cues	4.11	0.81	3.86	0.85	4.75	0.46	= .025	2-3
adapted their tone of voice appropriately to the situation	4.53	0.70	4.00	0.71	4.63	0.52	= .008	1-2
used their facial expressions adequately	4.28	0.86	3.95	0.81	4.88	0.35	= .024	2-3
used their gestures adequately	3.79	1.11	3.57	1.21	4.88	0.35	= .015 = .027	1-3 2-3
was distracted or preoccupied with other things	1.54	1.11	1.43	0.81	1.0	0.0	> .05	-

### Empathy in the patient-physician communication

The students reported being moderately empathic (M = 4.06±0.31). The tutors saw the medical students who took the medical history as empathic (M = 5.52±1.23). The SP agreed that the medical students in the physician’s role behaved empathically (M = 6.16±1.11). However, the tutors and SPs differed significantly on two points, including “taking patient’s perspective (M_tutors_ = 5.57±1.25; M_simulated patient_ = 6.5±0.76; *p* < .05) and “being interested in patient’s daily life (M_tutors_ = 5.48±1.12; M = 6.50±0.76; *p* < .05).

### Study environment

A total of 76.1% of the medical students participated in the online tutorial at their desks, while 15.4% took part in a designated office room; 83.5% of them experienced no technical difficulties. Neither the tutors (80.9%) nor the SPs (100.0%) faced technical difficulties. Furthermore, the medical students reported that they were not disturbed by external factors, such as children or noise (M = 3.07±2.20). However, 33.3% admitted that they were distracted by other factors like email or social media.

### Learning success

There was a significant increase in the medical students’ learning success over the three time points that were considered. They could name the relevant aspects more correctly as the course progressed and reached the highest score at time point 2, as shown in Table 2.

**Table 2:** Learning success over the three time points

Time points (T)	N	Learning success		Cohen’s d	
		Mean	SD		
1	128	3.65	2.61	T1/T2	0.36
2	96	4.55	2.42	T2/T3	0.41
3	53	5.53	2.30	T1/T3	0.74

## Discussion

This study investigated the efficacy of online tutorials in a communication course by focusing on interaction, verbal and nonverbal communication, and empathy. The students’ learning success was also assessed over the duration of the communication course.

### Principal results

The results showed that the interaction in the online setting was good, with both students and tutors reporting that they found each other likeable. Moreover, the students reported that they learned a lot during the course; this was confirmed by the assessment of their learning success, which increased throughout the duration of the course. The nonverbal and verbal communication in the encounter with the SP was also rated as high by all three groups, including students, tutors, and SPs; the SPs showed the highest scores, followed by students and then tutors. SPs as well as tutors saw the medical students as empathic in their role as physician. In general, no technical difficulties or limitations were reported by the three groups. In the evaluation of the online tutorial, the students agreed that it was very helpful and that they would recommend it to their fellow students, although they preferred a face-to-face version.

### Interaction

The students and tutors agreed that the level of interaction was good in the online tutorial. Similar to previous studies that investigated PAL in a face-to-face setting, the results showed that the tutors used their knowledge to help the students and behaved empathically [6, 7, 20, 21]. Despite the online setting, students could express their difficulties to the tutor and reported that they learned a lot in the online tutorial.

### Nonverbal and verbal communication and empathy

In the patient–physician encounter, students, tutors, and SPs rated the nonverbal and verbal communication, such as maintaining eye contact, as high. When compared among the three groups, SPs rated nonverbal and verbal communication as the highest; this was likely because they were the most involved in the encounter and focused more on the students’ nonverbal and verbal communication cues in order to give constructive feedback to the students afterwards [22, 23]. Although Gaur et al. [24] suggested that nonverbal and verbal communication cues, such as facial expressions, might be compromised to some extent in online teaching, this study showed that they can be preserved in a synchronous online teaching course. This result is consistent with the findings of Jiang et al. [25], who recommended the implementation of SPs into online learning during the pandemic. However, to ensure accurate nonverbal and verbal communication cues in online learning, participants are not allowed to be distracted or preoccupied with other things; this could be a potential issue because one-third of our participating students admitted to experiencing such distractions.

Regarding empathy in the patient–physician encounter, SPs as well as tutors agreed that the students behaved empathically in the role of physician. Again, the SP agreed more strongly when asked whether the student was able to consider the patient’s perspective and showed interest in the patient’s daily life; this might be strengthened by the students’ use of nonverbal and verbal communication cues [26].

### Learning success

As reported by previous studies, learning was successful; in the online tutorial, students were able to learn the relevant elements of a patient’s psychosocial medical history and how to record such a history [14, 15]. Despite the online setting, the tutors acted effectively in the digital environment and might be as good as professional lecturers [27–29].

### General evaluation and technical difficulties

Students as well as tutors disagreed that they preferred the online format in comparison to an in-person teaching situation. However, they found it very helpful under the circumstances given and would recommend it to fellow students.

No technical difficulties were reported by the three groups. Several studies have reported that it was important that the online platform used for online teaching worked well and that the participants were able to handle the technical aspects of the system [13, 25]. The students’ ability to handle the platform and a working video system contributed to the efficacy of the online tutorial [13, 25].

### Strengths and limitations

To the best of our knowledge, this study presents the first investigation of interaction and nonverbal and verbal communications in an online tutorial setting. The results demonstrate that communication skills can be taught successfully in an online tutorial and that students perceive this kind of learning as helpful. However, this study is limited by the fact that teaching communication skills in an online setting might be easier than teaching manual skills. Thus, future research could investigate the efficacy of online tutorials when teaching manual skills. Furthermore, the numbers of tutors and SPs are smaller than the numbers of students, which makes group comparisons difficult. However, we validated the significant comparisons by using nonparametric tests and reported the means. Hence, the groups are comparable.

## Conclusions

Interaction and nonverbal and verbal communication can take place in an online format, indicating that online tutorials can be effective. However, ensuring their success requires, among other things, the elimination of technical difficulties and the provision of assistance to the participants to avoid distractions. The tutors must be trained in advance in handling the technique and should be made aware of the specifics applicable to online teaching. The implementation of SPs might enhance the efficiency of synchronous online learning as it enhances the simulation of a patient–physician encounter. Thus, online tutorials might be a valuable amendment to medical education. Although online tutorials will certainly outlive the pandemic, face-to-face training will always remain the primary cornerstone in medical education. Nonetheless, the digital version of traditional PAL is a valuable modification to medical education.

## Data Availability

The datasets used and/or analysed during the current study are available from the corresponding author on reasonable request
